# Fibroblast growth factor-23 and calcium phosphate product in young chronic kidney disease patients: a cross-sectional study

**DOI:** 10.1186/1471-2369-14-39

**Published:** 2013-02-17

**Authors:** Abeer Yasin, Daisy Liu, Luan Chau, Joaquín Madrenas, Guido Filler

**Affiliations:** 1 Department of Pediatrics, Children’s Hospital, London Health Science Center, University of Western Ontario, 800 Commissioners Road East, London, Ontario, N6A 5W9, Canada; 2 Department of Microbiology and Immunology, and Medicine, and Center for Human Immunology, Schulich School of Medicine & Dentistry, University of Western Ontario, London, Ontario, N6A 3K7, Canada; 3 Department of Pathology and Laboratory Medicine, DSB 4044, Schulich School of Medicine and Dentistry, University f Western Ontario, London, Ontario, N6A 3K7, Canada; 4 Department of Microbiology & Immunology, McGill University, Room 511, Duff Medical Building, Montreal, Quebec, H3A 2B4, Canada

**Keywords:** Chronic kidney disease, Fibroblast growth factor 23, Parathyroid hormone, Renal osteodystrophy, Calcium phosphate product

## Abstract

**Background:**

Fibroblast growth factor-23 (FGF-23), a novel marker of bone disease in chronic kidney disease (CKD) has been shown to correlate with vascular calcifications. We aimed to describe the effect of the calcium phosphate product (Ca*P) on FGF-23 concentrations in children and young adults without confounding cardiovascular disease.

**Methods:**

Pediatric and young adult patients with CKD stages I-V were recruited in this cross sectional study to measure FGF-23, cystatin C, vitamin D-metabolites and other serum markers of bone metabolism. FGF-23 levels were determined with an enzyme-linked immunosorbent assay. The association between FGF-23 and (Ca*P) was assessed using non-parametric methods. Patients were divided into two age groups, less than 13 years of age and greater than 13 years of age.

**Results:**

This cross-sectional study measured serum FGF-23, in 81 patients (42 females, 51.9%) at London Health Sciences Centre, aged 2 to 25 years, with various stages of CKD (Cystatin C estimated glomerular filtration rate, eGFR=10.7-213.0 ml/min). For the whole entire group of patients, FGF-23 levels were found to correlate significantly with age (Spearman r= 0.26, p=0.0198), Cystatin C eGFR (Spearman r=−0.40 p=0.0002), CKD stage (Spearman r=0.457, p<0.0001), PTH (Spearman r=0.330, p=0.0039), ionized calcium (Spearman r=−0.330, p=0.0049), CysC (Spearman r= 0.404, p=0.0002) and 1,25-dihydroxyvitamin D (Spearman r=−0.345, p=0.0034) concentrations. No significant correlation was found between FGF-23 levels and calcium phosphate product (Spearman r= 0.164, p=0.142). Upon classification of patients into two age groups, less than 13 years of age and more than 13 years of age, correlational results differed significantly. FGF-23 correlated with CysC eGFR( Spearman r= −0.633, p<0.0001), CKD stage (Spearman r=0.731, p<0.0001), phosphate (Spearman r= 0.557, p<0.0001), calcium phosphate product (Spearman r=0.534, p<0.0001), 125(OH)2 Vit D (Spearman r=−0.631, p<0.0001), PTH (Spearman r= 0.475, p=0.0017) and ionized calcium (Spearman r= −0.503, p=0.0015) only in the older group. The relationship between FGF-23 and Ca*P for the older group could be expressed by the exponential model FGF-23= 38.15 e^0.4625Ca*P^.

**Conclusion:**

Abnormal values of FGF-23 in adolescents and young adults with CKD correlate with Ca* P in the absence of vascular calcifications, and may serve as a biomarker for the risk of cardiovascular calcifications.

## Background

Fibroblast growth factor-23 (FGF-23) is a phosphaturic hormone that increases in early chronic kidney disease (CKD) before abnormalities in serum calcium, phosphate, or parathyroid hormone (PTH) become apparent [1,2]. FGF-23 is thought to be produced by altered osteocyte function in early CKD [3] and is elevated in patients with end-stage kidney disease. FGF-23 has been linked with mortality, vascular calcification, markers of bone turnover, and left ventricular hypertrophy [4]. Cardiovascular disease is a major cause of morbidity and mortality in adult patients with end-stage renal disease receiving maintenance dialysis [5]. Cardiovascular mortality is also a significant issue in children and young adults with kidney disease, largely due to vascular calcifications in the media of vessels [6,7]. As higher phosphate levels are associated with vascular calcifications, several studies have examined the role of serum FGF-23 levels in phosphate metabolism and vascular calcifications. Calcium phosphate products (Ca*P) >55 mg/dl are independent predictors for coronary calcifications [8]. However, there is no clear link between FGF-23 levels and vascular calcifications. One study suggests that FGF-23 concentrations in blood are not associated with aortic calcifications [9]. Other studies, however, have clearly linked vascular calcifications with FGF-23 levels as an independent risk factor, even across all CKD stages [10]. Certainly, vascular calcification can be due to multiple etiologies such as hypertension, hypercholesterolemia, as well as calcium deposits; however, in children, the calcifications are mostly related to Ca*P products and CKD [6]. In fact, children and young adult patients with calcifications on dialysis have higher serum phosphorus concentrations and a higher calcium–phosphorus ion product in serum [11]. Desjardins et al. suggest that plasma FGF-23 is an independent biomarker of vascular calcification in patients with various CKD stages, including early stages [10]. FGF-23 has also been associated with endothelial dysfunction [12,13].

In addition to increased FGF-23 production by osteocytes [3], FGF-23 concentrations may also rise because of accumulation in the serum secondary to decreased glomerular filtration. FGF-23 is a small molecular weight molecule, similar to that of Cystatin C (CysC), which also accumulates in serum in patients with decreased renal clearance [14]. Elevated FGF-23 levels have been reported to suppress 1-alpha hydroxylase, worsen vitamin D deficiency, and contribute to secondary hyperparathyroidism [15]. Recently, there have also been reports that the renin-angiotensin-aldosterone system (RAAS) interacts with FGF-23 [16]. Few studies, however, have reported on the prevalence of FGF-23 and other markers of bone mineral metabolism disturbances in children with CKD [17,18]. There are many proposed mechanisms that link FGF-23 with cardiovascular morbidity and mortality. However, there is no data on the relationship between FGF-23 and the calcium phosphate product (Ca*P) in children and young adults, where the confounding factors associated with later age are less present.

The aim of the current study was to assess the relationship of FGF-23 with the Ca*P, CKD related mineral bone abnormalities, and the prevalence of therapies to correct them in a representative cohort of children and young adults with CKD who do not have obvious vascular calcifications on planar x-ray. We were curious to determine whether FGF-23 would correlate with the Ca*P in the absence of vascular calcifications.

## Methods

### Study population

After obtaining ethical approval from the University of Western Ontario Research Ethics Board (REB#16962E), we recruited 81 pediatric and young adult patients without evidence of cardiovascular disease in a cross-sectional study. All patients between the ages of 2 and 40 years with CKD stage I-V at London Health Sciences Centre, London, Ontario, were eligible. Written and informed consent were obtained for each patient. Patients with known vascular calcifications on chest x-rays were not considered; in addition, renal transplant recipients were excluded from this study.

In addition to routine blood work that was regularly obtained for the monitoring of the CKD, we obtained serum for FGF-23 and CysC levels in each case. We also measured phosphate, calcium, ionized calcium, serum albumin and total protein, bicarbonate, vitamin D metabolites (1, 25-dihydroxy- and 25-hydroxyvitamin D), and intact PTH levels, and urinary calcium to creatinine ratio, using standard laboratory tests. CysC eGFR was calculated using the “Filler-formula” [19,20]. Serum PTH concentrations were assayed by a solid-phase, two-site chemiluminescent enzyme-labelled immunometric assay (Immunlite 2000 Intact PTH from Diagnostic Products Corporation, Los Angeles, CA, USA). Ca*P was the simple product of the total serum calcium and the serum phosphate.

### Analytical validation of FGF-23 assay

Serum FGF-23 levels were determined with a sandwich enzyme-linked immunosorbent assay (ELISA) system using two kinds of monoclonal antibodies requiring the simultaneous presence of both the N-terminal and C-terminal portions of FGF-23 (Kainos Laboratories, Inc., Tokyo, Japan; Millipore, St. Charles, Missouri, USA) following the manufacturers’ instructions. In each antibody-coated well, 50 μL of serum sample with 50 μl of assay diluent were added to each well. The plate was then incubated at room temperature for 2 hours on a plate mixer. The plate was washed 4 times and incubated with FGF-23 conjugate mixing for 1 hour at room temperature. After another 4 washes, substrate was added and allowed to develop for 30 minutes. The signal was read in a microplate reader at absorbance 450 nm within 10mins [21]. Inter-assay and intra-assay coefficient of variation were 5.0 and 3.0%, respectively. Cystatin C was measured using the Siemens Healthcare nepholometric assay (PETIA) on a BN-Prospec platform (Dade-Behring) [19,22].

### Data analysis

Wherever possible, simple descriptive statistics were used. Contiguous data were tested for normality using the Shapiro-Wilk normality test. Normally distributed data were analyzed using parametric methods (mean, standard deviation, t-test, Pearson correlation), otherwise nonparametric methods were used (median, 25^th^ percentile, 75^th^ percentile, Mann Whitney t-test and Spearman rank correlation). All statistical analysis was performed using the statistical software GraphPad Prism, version 5.0 (GraphPad Inc, San Diego, CA, U.S.A). A p-value of < 0.05 was considered statistically significant.

## Results

**Figure 1:**
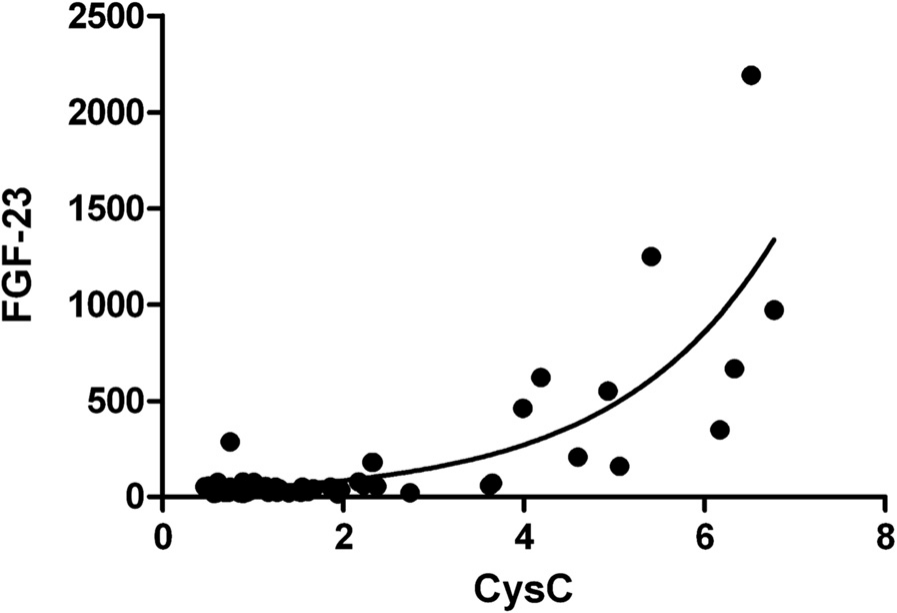
The relationship between FGF-23 concentrations and phosphate and Ca* P product. There was a significant correlation between FGF-23 and both phosphate (top, Spearman r=0.557, p < 0.0001) and Ca*P (bottom, Spearman r=0.534, p<0.0001) that followed an exponential growth pattern only in the group of patients older than 13 years of age. The exponential model could be described as FGF-23= 29.93 e^1.151P^ in the case of phosphate and FGF-23= 38.15 e ^0.4625Ca*P^ in the case of calcium phosphate product with regression coefficients of 0.8758 and 0.7819, respectively.

A total of 81 patients were included in the study. Median age was 13.3 years (25^th^ percentile 8.0, 75^th^ percentile 17 years), and 42 patients (51.9%) were female. Out of the 81 patients, 4 had hereditary cystic diseases (autosomal dominant and recessive polycystic kidney disease), 14 had tubular disorders including cystinosis, 34 had glomerular disorders (in particular, focal and segmental glomerulosclerosis), 17 had obstructive uropathy, and 12 had renal dysplasia. The distribution of the underlying diagnoses is given in Table 1, and while there was variability among the various CKD stages, this did not reach statistical significance. Forty four patients had CKD stage I (Cystatin C-based estimated glomerular filtration rate (eGFR) >90 mL/min/1.73 m^2^), 11 patients had CKD stage II (eGFR 60 to 89.9 mL/min/1.73 m^2^),13 patients had CKD stage III (eGFR 30 to 59.9 mL/min/1.73 m^2^) and 13 patients had a eGFR <30 mL/min/1.73 m^2^. As there were only 6 patients who were on dialysis, CKD stage IV and V were combined. The patients’ characteristics and median GFR, and the proportion of renal acidosis, hypertension, and renal osteodystrophy, along with the respective treatments, are summarized in Table 2. There was a significant association between the CKD stages and all parameters in Table 2 except for hypertension, which may have been confounded by the widespread use of angiotensin II converting enzyme inhibitors (ACE) or angiotensin II receptor blockers (ARB). There was also a progressive decline between GFR stage and 1,25-dihydroxyvitamin D concentrations, but interestingly, no changes in the 25-hydroxyvitamin D concentrations. All parameters in Table 3 were not normally distributed. To assess for any correlations between eGFR and other variables under study for the entire group of patients we performed the non-parametric spearman rank correlation analysis. Similar analyses were conducted for the two age groups of less than 13 years and greater than 13 years of age. Interestingly, FGF-23 correlated with CysC eGFR (negatively and significantly) but did not correlate with either phosphate or calcium phosphate product for the entire group of patients. This was despite of a similar eGFR range. For the younger group of age less than 13 years, FGF-23 did not correlate with CysC eGFR, phosphate or Ca*P while it correlated negatively and significantly with CysC eGFR, positively and significantly with phosphate and calcium phosphate product for the older group of patients aging 13 years and above (Figure 1). CysC eGFR was found to correlate positively and significantly with calcium, 1,25-dihydroxyvitamin D levels, ionized Ca and negatively with FGF-23, phosphate, 25-hydroxyvitamin D, alkaline phosphate and PTH for the entire group of patients. CysC eGFR correlations with bone markers differed when considering age groups. For the younger group of patients CysC eGFR correlated only with 1,25-dihydroxyvitamin D levels, PTH and ionized Ca and for the older group it correlated with FGF-23, Phosphate, Ca*P, 1,25-OH Vitamin D levels, alkaline phosphatase, PTH and ionized calcium only. X-rays were performed in 32 of the patients, with 100% of patients in the two highest stages of CKD. None showed calcifications. Table 3 summarizes the correlational analysis results.

**Table 1:** Breakdown of the various diagnoses groups overall and by CKD stage

**Diagnosis**	**Number**	**Overall %**	**% CKD stage I**	**% CKD stage II**	**% CKD stage III**	**% CKD stage IV+V**
Cystic	4	4.9%	2.3%	0.0%	7.7%	15.4%
Tubular	14	17.3%	20.5%	27.3%	15.4%	0.0%
Glomerular	34	42.0%	59.1%	36.4%	7.7%	23.1%
Uropathy	17	21.0%	13.6%	18.2%	23.1%	46.2%
Renal dysplasia	12	14.8%	4.5%	18.2%	46.2%	15.4%

**Table 2:** Patient characteristics, glomerular filtration rate (GFR), renal acidosis, use of corticosteroids, hypertension and renal osteodystrophy by CKD stage

**CKD stage**	**CKD stage I**	**CKD stage II**	**CKD stage III**	**CKD stage IV+V**
Number of patients	44	11	13	13
% of total patients	54.3%	13.6%	16.0%	16.0%
Median GFR (IQR 25th-75th percentile)	141.3 (105.1-161.9)	71.3 (65.8-82.8)	43.5 (36.4-5.9)	15.3 (11.7-20.4)
**Renal acidosis**				
Overall treated	11.4%	0.0%	30.8%	23.1%
Treated with bicarbonate (%)	2.3%	0.0%	23.1%	23.1%
Treated with potassium/sodium citrate (%)	9.1%	0.0%	7.7%	0.0%
**Hypertension**				
Patients on any BP meds (%)	54.5%	72.7%	46.2%	76.9%
Patients on ACE-I or ARB (%)	43.2%	72.7%	53.8%	46.2%
Patients on non-ACE-I (diuretics, Ca-channel blocker etc.) (%)	18.2%	27.3%	15.4%	53.8%
Patients on multiple therapies (%)	22.7%	36.4%	15.4%	23.1%
**Steroid therapy**				
% of patients on corticosteroid medications	18.2%	18.2%	7.7%	7.7%
**% Renal osteodystrophy treated with**				
calcium containing phosphate binders	0.0%	9.1%	15.4%	46.2%
non-calcium containing phosphate binders	0.0%	0.0%	0.0%	15.4%
vitamin D analog	6.8%	9.1%	0.0%	46.2%
active vitamin D analog	0.0%	9.1%	30.8%	53.8%
Abnormally high PTH (N=1.6-6.9 pmol/L)	6.8%	36.4%	69.2%	84.6%
Abnormally high FGF-23 (N=10-50 pg/mL)	13.6%	27.3%	53.8%	92.3%
Abnormally high phosphate (N=0.80-1.33 mmol/L)	40.9%	45.5%	15.4%	84.6%

IQR=Inter quartile range, %=percent, BP=blood pressure, PTH=parathyroid hormone, FGF-23=fibroblast growth factor 23, N=normal.

**Table 3:** Spearman rank correlational analysis of bone markers and CysC eGFR as well as FGF-23 for the group of all patients, group of <13 yrs and group of > 13 yrs of age

**Correlations of CysC eGFR**	**Correlation coefficient**	**P-value**	**Correlations of FGF-23**	**Correlation coefficient**	**P-value**
❖ ***All patients*** FGF-23	−0.4041	<0.0001	❖ ***All patients*** CysC eGFR	−0.4041	0.0002
CKD Stage	−0.8939	0.7688	CKD Stage	0.4574	< 0.0001
Calcium	0.0332	0.0090	Calcium	−0.2020	0.0705
Phosphate	−0.2887	0.0083	Phosphate	0.1791	0.1097
Ca*P	−0.2914	0.3484	Ca*P	0.1645	0.1422
25-OH Vit D	−0.1062	<0.0001	25-OH Vit D	0.0554	0.6253
125(OH)2 Vit D	0.5926	0.0241	125(OH)2 Vit D	−0.3455	0.0034
Alk Phosph	−0.2603	0.0002	Alk Phosph	−0.0858	0.4644
PTH	−0.5758	0.0002	PTH	0.3298	0.0039
ionized Ca	0.4278	<0.0001	ionized Ca	−0.3301	0.0049
❖ ***< 13 yrs***			❖ ***< 13 yrs***		
FGF-23	−0.0798	0.6388	CysC eGFR	−0.0798	0.6388
CKD Stage	−0.8850	<0.0001	CKD Stage	0.1054	0.5348
Calcium	0.0684	0.6873	Calcium	−0.1727	0.3067
Phosphate	0.0649	0.7029	Phosphate	−0.0324	0.8490
Ca*P	0.0605	0.7221	Ca*P	−0.0440	0.7960
25-OH Vit D	−0.3134	0.0589	25-OH Vit D	0.1307	0.4405
125(OH)2 Vit D	0.4281	0.0163	125(OH)2 Vit D	0.1203	0.5193
Alk Phosph	−0.2764	0.1080	Alk Phosph	−0.1290	0.4602
PTH	−0.4727	0.0048	PTH	0.1235	0.4864
ionized Ca	0.4468	0.0081	ionized Ca	−0.2085	0.2366
❖ ***> 13 yrs***			❖ ***> 13 yrs***		
FGF-23	−0.6330	<0.0001	CysCeGFR	−0.6330	<0.0001
CKD Stage	−0.8975	<0.0001	CKD Stage	0.7309	<0.0001
Calcium	0.0135	0.9305	Calcium	−0.1796	0.2435
Phosphate	−0.5558	<0.0001	Phosphate	0.5570	<0.0001
Ca*P	−0.5618	<0.0001	Ca*P	0.5343	<0.0001
25-OH Vit D	0.0400	0.7990	25-OH Vit D	0.0032	0.9835
125(OH)2 Vit D	0.6888	<0.0001	125(OH)2 Vit D	−0.6306	<0.0001
Alk Phosph	−0.4289	0.0058	Alk Phosph	0.2834	0.0764
PTH	−0.6071	<0.0001	PTH	0.4747	0.0017
ionized Ca	0.4592	0.0042	ionized Ca	−0.5034	0.0015

**Figure 2:**
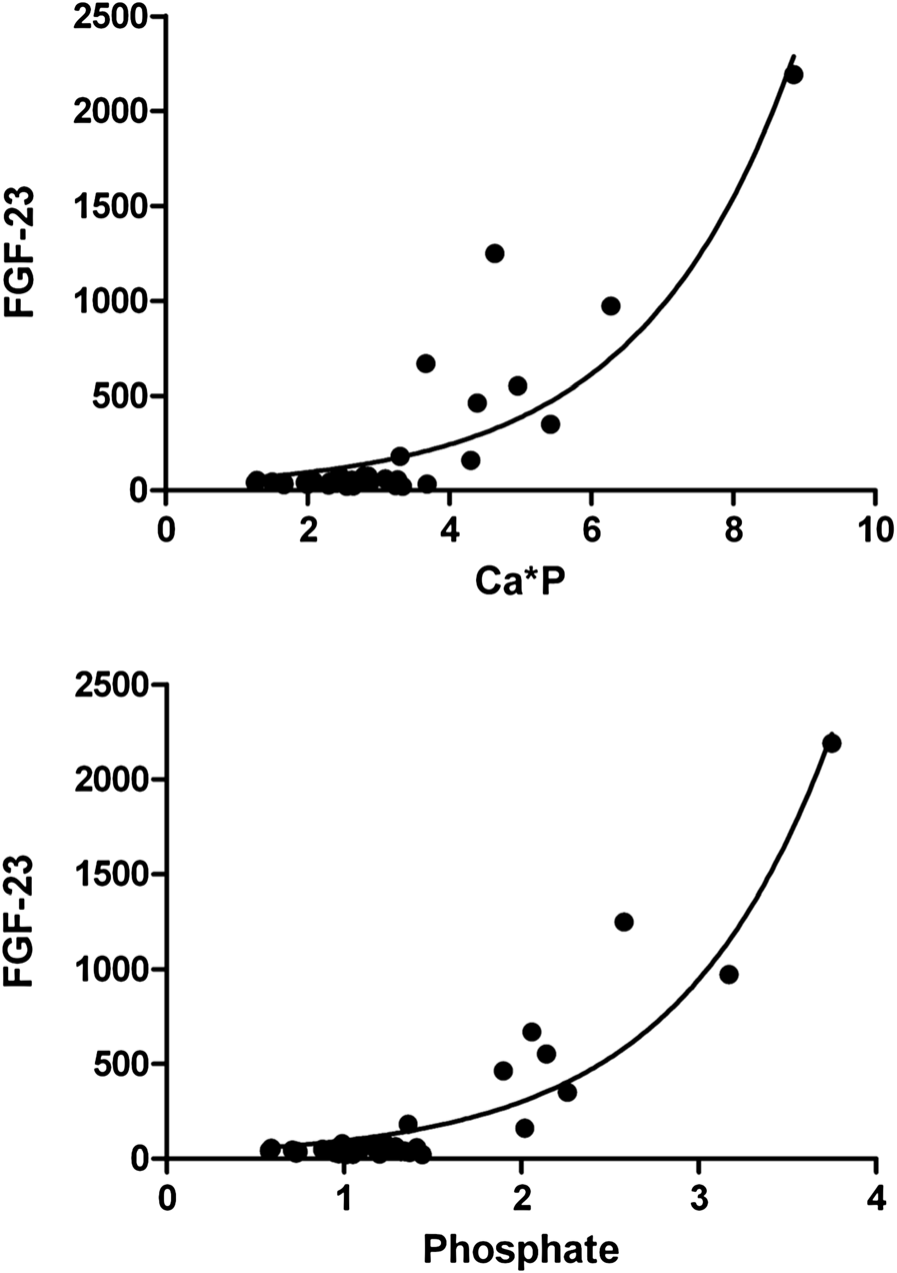
**There was a significant correlation (Spearman r=0.404, p<0.0001) between both markers, FGF-23 and CysC, for the whole group of patients that followed an exponential growth pattern.** The exponential model could be described as FGF-23= 27.10 e ^0.5759CysC^ with a regression coefficient of 0.6660.

Total calcium dropped slightly in CKD stage IV and V, while phosphate rose to abnormal values in CKD stage IV and V as did the intact PTH concentrations. However, noticeably abnormal values of 1,25-dihydroxyvitamin D and FGF-23 concentrations were already noted in patients with CKD stage III. The median values of calcium, phosphate, 25-hydroxyvitamin D, 1,25 dihydroxyvitamin D, alkaline phosphatase, intact PTH and FGF-23 concentrations are given in Table 4. While one cannot extrapolate from a cross-sectional study on the longitudinal evolution of mineral bone disease laboratory markers, it appears that the drop in the 1,25-dihydroxyvitamin D concentrations is more prevalent in patients with CKD stage III when compared to all other biochemical bone markers. FGF-23 correlated significantly with CysC (Figure 2 and Table 4).

**Table 4:** Bone parameters and CKD stage

**CKD stage**	**Calcium [mmol/L]**	**Phosphate [mmol/L]**	**25-OH vitamin D [nmol/L]**	**1,25 (OH)**_**2**_**vitamin D [pmol/L]**	**Alkaline phosphatase [U/L]**	**intact PTH [pmol/L]**	**FGF-23 [pg/mL]**
**CKD stage I**	2.32 (2.2-2.4)	1.27 (1.1-1.4)	61 (48.5-86.5)	82 (70.5-100.5)	153 (85.0-216.5)	3.8 (2.8-4.8)	39.4 (30.9-47.2)
**CKD stage II**	2.39 (2.3-2.4)	1.19 (1.2-1.4)	45 (44–91)	57 (46–73.5)	153 (96–283)	4.4 (3.3-7.8)	45.2 (24.3-51.6)
**CKD stage III**	2.36 (2.3-2.4)	1.22 (1.2-1.3)	64 (47–101)	53.5 (41–64.2)	218 (122.5-254)	8.8 (7–9.3)	52.8 (28.9 – 102.8)
**CKD stage IV+V**	2.22 (2.1-2.4)	2.06 (1.7-2.3)	58.5 (47.5-127.2)	17 (12–32.5)	188 (102–261)	44.4 (9.7-79.1)	461.1 (158.4 – 666.8)

Data are given in median and interquartile range (25^**th**^ and 75^th^ percentile).

## Discussion

The objective of the current study was to describe the relationship of FGF-23 values to Ca*P in children and young adults with CKD and no evidence of vascular calculations. The prevalence of complications worsened with CKD stage, similar to our previously published study on 366 children [23]. However, the previous study did not include data on FGF-23 and a detailed look at markers of bone mineral disease in CKD patients. The novel finding is the correlation of FGF-23 levels with the Ca*P product, which has not previously been reported in children, but interestingly only in adolescents and young adults.

Many of the relationships described in this manuscript have been reported previously by Magnusson et al. [24], Bacchetta et al. [17] and Srivaths et al. [18]. Magnusson et al’s study is the only prospective study, but may have been limited by the study’s small sample size (n=13). Several other studies have explored the relationship between FGF-23 and the correlation with other bone markers in paediatric CKD patients [14,25-27], but they have looked at smaller and more limited population of CKD patients – for instance, advanced CKD patients [25] or renal transplant patients [27,28]. This current study is more comprehensive, including CKD patients from all stages and varying aetiologies, and examines FGF-23 and its relationship with other bone markers across CKD stage.

The inverse relationship between FGF-23 and eGFR has been demonstrated in many studies, both in the pediatric and adult population [1,14,24,29]. The elevated FGF-23 levels observed in CKD have been explained by both increasing production by altered osteocyte function [3,19] and by accumulation secondary to decreased renal clearance, as FGF-23 is a low molecular weight protein that is freely filtered across the glomeruli [14]. The exact contribution of each to this process is unknown.

The main question of the study was the assessment of the effect of the Ca*P on the FGF-23 concentrations, which the literature only demonstrated in adults [8,10]. Our data would suggest that FGF-23 is associated with calcium phosphate metabolism disorders, and not necessarily with aortic calcifications, although we have to acknowledge the limitation that only 32 patients had a chest x-ray. With this limitation, our data would support the interpretation by Kojima et al. [9]. Of course it is not very surprising that FGF-23 levels correlated with Ca*P, as they correlated with phosphate, whereas calcium levels changed only mildly with increasing FGF-23 levels. Nonetheless, Ca*P has emerged as a key risk factor for cardiovascular calcifications, especially when assessing the area under the time concentration curve over time. FGF-23 levels may provide an additional marker of the morbidity and mortality of Ca*P as it is associated with endothelial dysfunction and cardiovascular outcome [13].

The main limitation of this study was the cross-sectional design. Although we demonstrated associations between several bone markers – including FGF-23 – and glomerular filtration rate, these associations may not be causative. A prospective, longitudinal study is required to further delineate the relationship between GFR and CKD-MBD parameters. Another limitation of our study was the use of estimated glomerular filtration rate by Cystatin C, rather than the use of GFR measurement by inulin, although the latter is time-consuming and not practical or well-suited to clinical practice and this cross-sectional design. Perhaps the most significant limitation is the lack of urinary phosphate measurements for the calculation of Tp/GFR, which may already be reduced at earlier stages of CKD than serum phosphate concentrations. Another limitation forms the fact that a small subset of patients received steroids, which may affect FGF-23 levels [17]. We also did not assess patients on their dietary phosphorus intake, which can contribute to phosphorus retention and bone mineral disroders with decreasing GFR.

## Conclusion

In conclusion, the current study describes FGF-23 in relationship to other CKD-MBD parameters in 81 CKD patients. As expected, the prevalence of abnormal findings and medians for 1,25-dihydroxyvitamin D, serum phosphate, PTH, serum calcium and FGF-23 changed with worsening kidney function. The study confirms the association of FGF-23 levels with the Ca*P only for patients older than 12 years of age.

## Abbreviations

ACE: Angiotensin converting enzyme inhibitor; ARB: Angiotensin II receptor blocker; Ca*P: Calcium phosphate product; CKD: Chronic kidney disease; CysC: Cystatin C; eGFR: Estimated glomerular filtration rate; FGF-23: Fibroblast growth factor-23; PTH: Parathyroid hormone.

## Competing interests

The authors declare that they have no competing interests.

## Authors’ contribution

AY performed the statistical analysis, conceived the idea of separating children from adolescents and young adults, and revised the manuscript, DL drafted the first version of the manuscript, participated in the design of the study and data collection, LC and JM carried out the immunoassay to measure serum FGF-23 levels. GF conceived of the study, designed and funded it, participated in its coordination, obtained ethics approval, and substantially edited the manuscript. All authors read and approved the final manuscript.

## Pre-publication history

The pre-publication history for this paper can be accessed here:

http://www.biomedcentral.com/1471-2369/14/39/prepub
